# Particle Filter-Guided Online Neural Networks for Multi-Target Bearing-Only Tracking in Passive Sonar Systems

**DOI:** 10.3390/s25185721

**Published:** 2025-09-13

**Authors:** Jianan Wang, Lujun Wang, Zhuoran Wang, Liang Xie, Huang Hu

**Affiliations:** 1 Science and Technology on Sonar Laboratory, Hangzhou 310023, China; wjnan1122@163.com (J.W.); w3016131@163.com (Z.W.); 13735702700@163.com (H.H.); 2 Hangzhou Applied Acoustics Research Institute, Hangzhou 310023, China; xieliangwork@163.com

**Keywords:** multi-target tracking, deep learning, passive sonar

## Abstract

This study proposes a novel method to address the instability issues in multi-target bearing-only tracking for passive sonar systems. Utilizing a particle filter-guided on-site training mechanism, the complex multi-classification task is simplified into binary classification (target vs. non-target) by assigning an independent tracker to each target. This enables simultaneous on-site training and deployment of the neural network for tracking. A hybrid CNN-BiLSTM network is constructed: the Convolutional Neural Network (CNN) enhances target feature extraction and non-target discrimination, while the Bidirectional Long Short-Term Memory (BiLSTM) models spatiotemporal dependencies. Their synergy improves trajectory continuity and smoothness. Under simulated conditions, the proposed method reduces the minimum required SNR for stable tracking to −31.78 dB, a significant improvement over the −29.69 dB required by pure particle filtering methods. The average tracking error is also reduced from 0.61° to 0.34°. Both simulations and sea trial data demonstrate that the method maintains stable tracking even during target trajectory crossings, significantly enhancing multi-target tracking accuracy in complex underwater acoustic environments.

## 1. Introduction

Sonar is a critical technology for marine exploration and anti-submarine warfare. Passive sonar systems [1], which do not actively emit signals, offer high concealment [2,3] and are widely used for underwater target detection by vessels [4]. Passive sonar bearing-only tracking is a key technique for obtaining target azimuth from multi-beam measurements, holding significant value in target localization. However, in complex real-world ocean environments, factors such as background noise [5] and intersecting target trajectories often lead to tracking instability or even mis-tracking, compromising tracking reliability.

The most fundamental acoustic tracking method is the maximum value tracker [6], which locates targets by iteratively detecting spectral peaks in local energy. While computationally simple, its performance heavily relies on target SNR and trajectory continuity, leading to significant degradation under strong noise or highly maneuvering targets. Current mainstream acoustic tracking methods primarily utilize two algorithmic frameworks: state estimation filters [7,8,9] and data association. The Kalman filter [10,11], a classical linear estimation algorithm, predicts target states via dynamic models and corrects them with observations. However, its linear motion assumption struggles with complex maneuvering scenarios. Particle filtering methods [12,13,14,15,16,17,18,19] can handle nonlinearity but still face accuracy challenges during multi-target crossing and occlusion. Data association methods [20,21,22] establish observation-to-track mappings using spatiotemporal metrics (e.g., distance, motion models). However, they are prone to identity confusion [23] during dense target encounters and exhibit strong dependence on prior knowledge [24].

The rise of deep neural networks (DNNs) offers a novel approach for complex acoustic pattern recognition [25,26,27], surpassing traditional methods in fields like speech processing and natural language understanding. Their application to sonar tracking presents dual advantages: First, neural networks circumvent strong assumptions about target motion models, making them more suitable for tracking complexly maneuvering targets. Second, they effectively extract deep-level features from raw sonar data. These features facilitate target classification [28,29] and enhance tracking accuracy, enabling more flexible handling of complex scenarios like multi-target trajectory crossings and occlusions while reducing reliance on prior knowledge.

Recent applications of deep neural networks in acoustic tracking have primarily focused on visual tracking for imaging sonar. Chen et al. [30] employed a Convolutional Neural Network (CNN) to address underwater low-contrast visual tracking challenges, while Shi [31] designed a dual-branch network to improve robustness against target deformation. For detection sonar, researchers have predominantly sought to integrate neural networks into traditional tracking algorithms, enhancing accuracy and real-time performance through structural improvements and parameter optimization. For instance, Ding [32] fused a backpropagation network with an Extended Kalman Filter (EKF) for online error correction. Other scholars have utilized neural networks to predict target motion trajectories for tracking, such as Yang et al. [33], who generated training data via motion modeling to track targets with multiple motion patterns. However, such methods primarily focus on motion features (trajectory, velocity, acceleration) and may fail with highly maneuverable targets.

Research directly utilizing neural networks for underwater target tracking remains scarce. Traditional neural network models require vast amounts of training data, which is difficult to obtain for marine targets, especially non-cooperative ones. This data scarcity leads to insufficient training, unstable model architectures, and large tracking errors. Another challenge is generalization: despite their strong learning capacity, the inherent complexity and instability of the marine environment introduce numerous interfering factors into sonar data, adversely affecting algorithm generalization.

This study aims to develop a novel and robust multi-target bearing-only tracking algorithm for passive sonar systems, enhancing tracking accuracy and stability—particularly under challenging conditions such as trajectory crossings and low SNR. Additionally, we seek to mitigate the heavy dependence on large, pre-collected training datasets that commonly constrains conventional neural network-based methods.

The innovative contributions of this paper are threefold:

1. Transformation of complex multi-classification into parallel binary classification tasks: Multi-target identification typically involves intricate relationships between target types, demanding high sample quantity and quality. By assigning a dedicated tracker (i.e., an independent parameter set) to each target, the complex many-to-many classification problem is transformed into simpler binary classification (target signal vs. non-target signal) for each tracker, significantly simplifying the learning process.
2. Design of a particle filtering method-guided on-site training mechanism: This not only overcomes the persistent bottleneck of data scarcity but also enhances the algorithm’s effectiveness across diverse marine environments and target types, improving model generalization.
3. Proposal of a spatiotemporal continuity-aware neural network architecture: Conventional CNNs effectively extract features distinguishing targets from noise but lack inherent temporal modeling capabilities, leading to trajectory jumps across consecutive frames. By integrating Bi-LSTM’s temporal modeling power with CNN, the proposed hybrid architecture improves trajectory continuity and tracking accuracy compared to single-network approaches, as validated experimentally.

Results demonstrate the proposed method’s significantly superior tracking accuracy under target trajectory crossing and low-SNR conditions compared to traditional passive sonar tracking techniques.

The paper is structured as follows: Section 2 introduces the fundamentals of bearing-only passive tracking and details the proposed algorithm framework and deep learning design. Section 3 presents experimental validation using simulated and sea trial data. Section 4 concludes the paper and discusses future work.

## 2. Materials and Methods

### 2.1. Fundamentals of Bearing-Only Passive Tracking

The core task of bearing-only multi-target tracking for passive sonar [34,35] is to reconstruct target motion trajectories from array-received signals, involving a three-stage information transformation process: array signal acquisition, beamforming, extracting trajectories from the Bearing-Time Record (BTR) [36].

#### 2.1.1. Array Signal Generation

**Figure 1:**
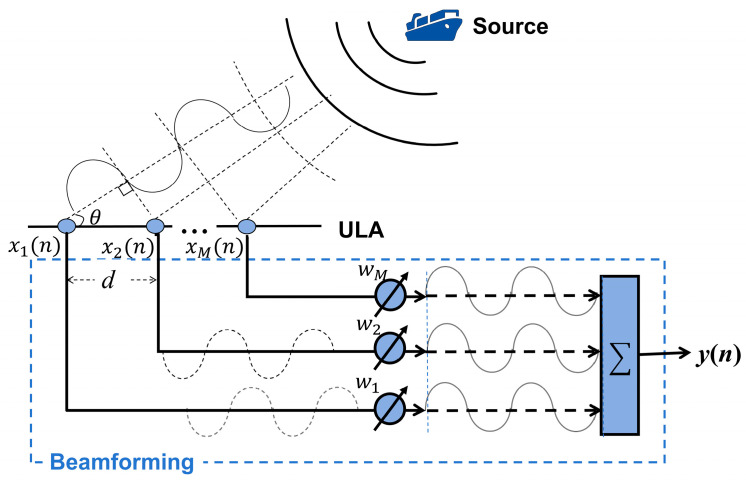
Beamforming process.

As shown in Figure 1, acoustic waves radiated from far-field targets propagate as plane waves to an M-element Uniform Linear Array (ULA) with element spacing d, incident angle θ, frequency f, and sound speed v. The time delay and phase shift of the m-th element relative to the reference element are

(1) $$\tau =\frac{dsin\theta }{v}$$

(2) $$\emptyset =\omega \tau =\frac{2\pi f\sin\theta }{v}=\frac{2\pi d\sin\theta }{\lambda }$$

The array-received signal can be expressed as

(3) x(t)=A(θ)s(t)+n(t)

where $A(\theta )=[a(\theta_{1}),\ldots ,a(\theta_{P})]$ is the steering matrix, and n(t) represents received noise.

(4) $$a(\theta )=[1,e^{-j2\pi d\sin\theta /\lambda },\ldots ,e^{-j2\pi (M-1)d\sin\theta /\lambda }{]}^{T}$$

#### 2.1.2. Beamforming

Addressing the broadband signal characteristics of practical sonar targets, this study employs a frequency-domain broadband beamforming method. First, the time-domain signals received by each array element are segmented, and a Fast Fourier Transform (FFT) is applied to each snapshot, yielding frequency-domain snapshot data:

(5) $$X(f_{k},t_{n})=FFT(x(t))$$

where $f_{k}$ denotes the k-th frequency bin and $t_{n}$ denotes the n-th time snapshot. Then, for each frequency bin $f_{k}$, the corresponding spatial spectrum is calculated:

(6) $$P(\theta ,f_{k},t_{n})=a^{H}(\theta ,f_{k})\hat{R}(f_{k})a(\theta ,f_{k})$$

Here, $\hat{R}(f_{k})$ is the estimated sample covariance matrix at frequency bin $f_{k}$, and $a(\theta ,f_{k})$ is the steering vector at $f_{k}$ (its elements depend on frequency and wavelength).

(7) $$a(\theta ,f_{k})={\left[{1,e}^{-j\frac{2\pi d\sin\theta }{\lambda_{k}}},\ldots ,\;e^{-j\frac{2\pi (M-1)d\sin\theta }{\lambda_{k}}}\right]}^{T}$$

Finally, the beam output powers from all frequency bins are combined via non-coherent integration (power summation) to obtain the total broadband beamforming power at azimuth θ and time snapshot $t_{n}$:

(8) $$P_{wide}(\theta ,t_{n})=\sum_{k=1}^{K}P(\theta ,f_{k},t_{n})=\sum_{k=1}^{K}a^{H}(\theta ,f_{k})\hat{R}(f_{k})a(\theta ,f_{k})$$

This process effectively fuses the bearing information of the broadband signal across different frequency components.

Beamforming provides the fundamental data for the BTR, with the former offering instantaneous bearing estimates and the latter exhibiting the continuity of target motion through time series.

#### 2.1.3. Classical Tracking Methods

##### Local Peak Detection (Maximum Value Tracking)

After one scan, the beam signal intensity in a specific direction corresponds to a grayscale level, with higher intensity yielding brighter pixels. By displaying the grayscale values corresponding to the beam output energy in each direction over successive time rows, the BTR is generated. The most fundamental method for target extraction on the BTR is peak detection.

Its core idea is to determine possible target bearings at each time snapshot by detecting local maxima in the beam energy spectrum $P_{wide}(\theta ,t_{n})$. Mathematically:

For time snapshot $t_{n}$, scan the azimuth angle θ (typically discretized into beam indices), seeking azimuth points $\theta_{max}$ satisfying

(9) $$\theta_{max}=argmax\left(P_{wide}\left(\theta ,t_{n}\right)\right),\;\;\theta \in [\theta_{max}-\Delta \theta ,\theta_{max}+∆\theta ]$$

That is, points with the highest energy $\theta_{max}$ within a small local neighborhood Δθ (typically several beamwidths). The energy $P_{wide}(\theta_{max},t_{n})$ at detected local maximum $\theta_{max}$ must exceed a preset detection threshold η to be confirmed as valid target points. Target points detected on adjacent snapshots are connected based on the nearest neighbor principle or simple motion smoothness assumptions to form estimated trajectories $\hat{\theta }(t)$.

This method is simple for engineering implementation, offers low computational complexity and excellent real-time performance, and requires no prior target knowledge. However, under low SNR conditions, the true peak of the target may fall below the detection threshold, leading to missed detections, or noise may exceed the threshold, causing false alarms. Furthermore, when two or more targets are close in bearing or near trajectory crossings, their energy peaks may merge into a single broader peak, or the sidelobe of a stronger target may mask a nearby weaker target. Maximum detection can only find one peak location, causing the trajectory of the weaker target to be erroneously connected to the peak of the stronger target.

##### Particle Filter Tracker

As a key implementation of nonlinear Bayesian estimation, the particle filtering method demonstrates unique advantages in underwater target tracking. Its core idea is to approximate complex probability distributions via Monte Carlo sampling, making it particularly suitable for state estimation under non-Gaussian noise.

Let the target state vector at time k be $\left\{x^{k};k\in N,x^{k}\in R^{n}\right\}$, where n is the state dimension, and the observation vector be $\left\{y^{k};k\in N,y^{k}\in R^{m}\right\}$, where ‘m’ is the observation dimension. The core problem of the particle filter tracker is to estimate the expected value of the target state $x^{k}$:

(10) $$E[f(x^{k})]=\int f(x^{0:k})p(x^{0:k}|y^{1:k})dx^{0:k}$$

where the posterior distribution $p(x^{0:k}|y^{1:k})$ is difficult to solve directly. The particle filtering method employs Monte Carlo approximation via importance sampling. The posterior is approximated by N particles:

(11) $$p(x^{0:k}|y^{1:k})\approx \frac{1}{N}\sum_{i=1}^{N}\delta \left(x-x_{i}^{0:k}\right)$$

Assuming $p(x^{0:k-1}|y^{1:k-1})$ is known, the posterior can be decomposed recursively using Bayes’ theorem and the Markov property of the target state:

(12) $$p(x^{0:k}|y^{1:k})\propto p(y^{k}|x^{k})\cdot p(x^{k}|x^{k-1})\cdot p(x^{0:k-1}|y^{1:k-1})$$

Introducing a proposal distribution $q(x^{0:k}|y^{1:k})$ that is easy to sample from, the expectation is rewritten as

(13) $$E[f(x^{k})]=\int f(x^{0:k})\frac{p(x^{0:k}|y^{1:k})}{q(x^{0:k}|y^{1:k})}q(\cdot )dx^{0:k}\approx \frac{1}{N}\sum_{i=1}^{N}{f(x}_{i}^{0:k})w_{i}^{k}$$

where $w_{i}^{k}$ is the normalized “weight” of particle i at time k, representing its “importance” in approximating the target probability density function. Substituting the weight formula yields the recursive form:

(14) $$w_{i}^{k}\propto \frac{p(y^{k}|x_{i}^{k})p(x_{i}^{k}|x_{i}^{k-1})}{q(x_{i}|x_{i}^{k-1},y^{k})}{\cdot w}_{i}^{k-1}$$

Weight calculation can be approximated by computing the unnormalized weight $W_{i}^{k}$ proportional to it, followed by normalization:

(15) $$W_{i}^{k}=p(y^{k}|x_{i}^{k})p(x_{i}^{k}|x_{i}^{k-1})\cdot w_{i}^{k-1}{,\;w}_{i}^{k}=\frac{W_{i}^{k}}{\sum_{i=1}^{N}W_{i}^{k}}$$

For the passive sonar bearing-only tracking scenario, the conventional approach uses the energy distribution of the BTR as the input and likelihood function for the particle filter tracker. The target state is defined as the bearing angle $\theta^{k}$, and its observation is directly taken from the BTR energy distribution $P(\theta ,t_{k})$. During initialization, the initial bearing $\theta^{0}$ is determined via energy threshold detection, and a particle swarm obeying a Gaussian distribution $N(\theta^{0},\sigma^{2})$ is generated. In the prediction stage, particles undergo random diffusion within a neighborhood around the previous estimated bearing ${\hat{\theta }}^{k-1}$. Weight updates directly utilize the BTR energy value as the likelihood measure:

(16) $$w_{i}^{k}\propto P(\theta_{i}^{k|k-1},t_{k})$$

Target bearing estimation is achieved via weighted average:

(17) $${\hat{\theta }}^{k}=\sum w_{i}^{k}\cdot \theta_{i}^{k|k-1}$$

The advantages of this method lie in its ability to handle nonlinear problems and explicitly model target motion through a state-space model. Therefore, when observations are temporarily missing (e.g., due to low SNR) but the trajectory remains continuous, motion model predictions maintain tracking continuity. However, the particle filter tracker also has limitations in trajectory crossing scenarios. When multiple target trajectories cross in bearing, particles tend to cluster towards the target with higher energy. After crossing and separation, the particle swarm may fail to correctly “split” back to their respective target trajectories, leading to mis-tracking.

### 2.2. Neural Network Tracking Method Based on On-Site Training

#### 2.2.1. Overall Algorithm Architecture

**Figure 2:**
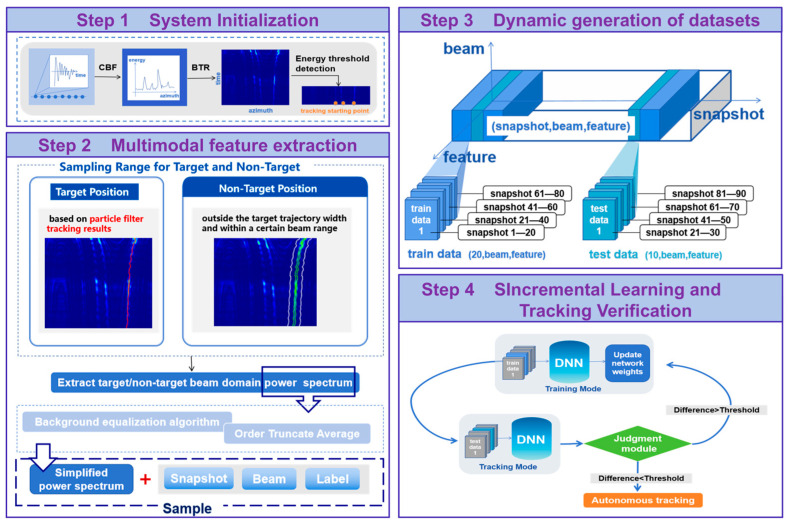
Overall algorithm architecture.

This study proposes a neural network-based bearing-only multi-target tracking algorithm for passive sonar, applying neural networks to detection sonar signal tracking. To address the challenge of scarce training data in practical applications, the algorithm employs an on-site learning mechanism combined with particle filter tracker to obtain initial training data. The overall architecture is shown in Figure 2; the specific implementation involves the following key steps:

Step 1 (System Initialization):

- Perform broadband beamforming on array signals to generate the BTR.
- Determine tracking starting points via automatic energy threshold detection.
- Launch particle filter trackers for initial trajectory estimation.

Step 2 (Multi-modal Feature Extraction):

- Determine target and non-target sampling regions based on particle filter tracker’s results.
- Extract beam-domain power spectrum features.
- Apply background equalization and Order Truncate Average (OTA) noise suppression to the beam-domain power spectrum.
- Incorporate timestamp (snapshot number) and spatial information (beam index) to construct feature vectors, and assign binary labels (target samples labeled as 1, background samples labeled as 0).

Step 3 (Dynamic Dataset Generation):

- Establish a temporal sliding window mechanism: Accumulate 10 consecutive snapshots to build a training set, then collect the subsequent 10 snapshots to form an independent test set.

Step 4 (Incremental Learning and Tracking Verification):

- Adopt an incremental training strategy to update deep neural network parameters.
- After each training iteration, update weights and perform autonomous tracking for the next 10 time snapshots.
- Execute a consistency check algorithm based on the particle filter tracker’s results.
- If the error exceeds the limit, return to training; otherwise, lock the current neural network parameters for subsequent tracking.

#### 2.2.2. Functional Module Design

1. Data Preprocessing Module

Performs broadband beamforming on time-domain signals acquired by the hydrophone array; generates the BTR and performs energy threshold detection to record initial tracking positions meeting the conditions.

2. Particle Filter Tracker Module

Executes particle filtering method-based tracking snapshot-by-snapshot, recording the time and bearing of targets.

3. Feature Extraction Module

Extracts beam-domain power spectra for targets and non-targets. The target sampling range is uniquely determined by the target position (time, beam) obtained from the particle filter tracker. To enhance discrimination, non-target sampling should occur outside the target trajectory width, estimated as the −3dB beamwidth relative to the target’s peak energy. Meanwhile, to maintain the balance between positive and negative samples and ensure training efficiency, the scope of non-target acquisition should not be excessively large. Therefore, we selected the maximum distance between the target under tracking and adjacent objects as the determination criterion. This adaptive sampling strategy, evolving with the target trajectory, effectively samples and learns interfering targets that may gradually approach and impact subsequent neural network classification.

The original power spectrum undergoes feature point compression via background equalization, followed by background noise suppression using the Order Truncate Average (OTA) algorithm, generating the final 1D power spectrum feature row vector. The snapshot number and beam index are appended to the row as spatiotemporal features. Finally, each sample is labeled (1 for target, 0 for non-target). Samples collected over 10 consecutive time snapshots are bundled into one training dataset.

4. Neural Network Module

To frequently verify learning effectiveness, a dual-mode (training/tracking) operational mechanism is designed. After training on a batch of data, the network automatically enters tracking mode. Parameters fed back from the decision module determine whether to continue training. In training mode, the network incrementally updates weights. In tracking mode, based on target motion inertia, beam-domain power spectra within ± 5 beams around the previous target bearing are input to the tracking neural network. The network outputs the bearing with the highest probability as the target location, and the result is stored.

5. Decision Module

Compares the difference between the target beam positions output by the particle filter tracker and the tracking neural network at the same time. After the tracking neural network outputs results for 10 consecutive snapshots, the decision module is activated. It calculates the tracking deviation between the two algorithms over these 10 snapshots, compares it to a preset threshold, and makes a decision on whether to activate autonomous neural network tracking.

This architecture effectively addresses the challenge of neural network training under small-sample conditions through a particle filter tracker-guided progressive learning strategy.

#### 2.2.3. CNN-BiLSTM Network Design

Although powerful models such as Siamese networks and Transformers have demonstrated excellent performance in visual tracking tasks, the features we expect the neural network to learn are derived from information-rich beamformed sonar signals rather than simple sonar imagery. On the other hand, their high computational cost hinders practical deployment in sonar tracking systems. We prioritize lightweight and efficient learning from sequential data over purely pursuing the highest accuracy. Therefore, we adopt a compact CNN-based architecture that balances learning capacity and operational speed. The core idea of Convolutional Neural Networks (CNNs) is to extract spatial features from data through convolutional operations. In the sonar target tracking task investigated in this paper, CNN is primarily used to extract the differences between targets and noise from one-dimensional power spectra. Through multi-layer convolution, it enhances robustness to signals under low signal-to-noise ratio conditions. However, CNN itself lacks the capability for temporal modeling, which leads to abrupt changes in its output when handling continuous-time tracking problems. Therefore, when processing sequential data, CNN serves as a preprocessing module within a hybrid model, extracting spatial features and transmitting them to temporal networks such as LSTM to compensate for this limitation.

Long Short-Term Memory (LSTM) networks can maintain long-term memory of important information over extended sequences, making them highly suitable for capturing long-term dependencies in temporal data [37,38,39,40]. In the context of sonar target tracking, LSTM can effectively model the motion trajectories of targets [41,42]. By employing Bidirectional Long Short-Term Memory networks (BiLSTM), the network can leverage both historical and future data simultaneously, thereby enhancing its temporal modeling capabilities.

This paper proposes a hybrid model that integrates CNN and BiLSTM. The first stage of the model employs a lightweight CNN architecture designed for efficient processing of hydroacoustic signal power spectra. The convolutional layers extract spatial features from the power spectra, effectively identifying patterns and relationships among different data points, thereby providing a solid foundation for subsequent time series analysis. The extracted features are then fed into a BiLSTM layer, which demonstrates exceptional performance in capturing temporal correlations within time series data. Since target trajectories often exhibit specific dynamic characteristics over time, the BiLSTM can analyze these time series, comprehend their intrinsic dynamics, and identify anomalies that deviate from normal trajectories. Through a hierarchical feature learning mechanism, this model achieves the organic fusion of local pattern perception and global temporal dependency modeling, offering a novel solution for multi-dimensional time series classification tasks.

**Figure 3:**
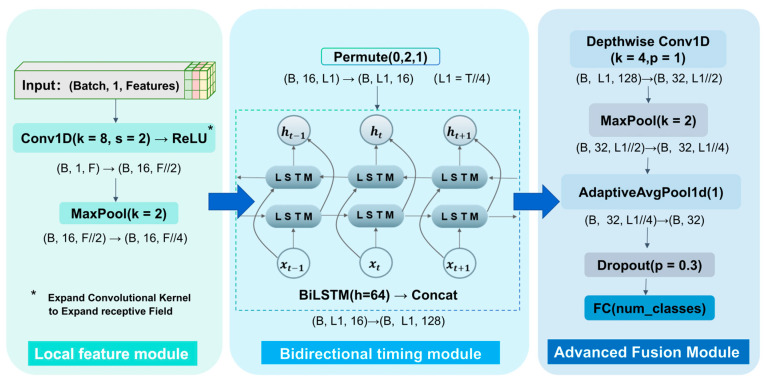
CNN-BiLSTM architecture.

The architecture adopts a modular design philosophy (as shown in Figure 3), comprising three core feature learning stages:

Local Feature Extraction Module

Employs a cascade structure of 1D convolutional layers (Conv1D: kernel_size = 8, stride = 2) and max-pooling layers to perform multi-scale feature abstraction on the input signal. Dilated convolutional kernels effectively expand the receptive field to capture physically meaningful short-term patterns (e.g., line spectra of acoustic signals). Output dimension: (batch_size, 16, L1), where L1 is the downsampled temporal length.

Bidirectional Temporal Modeling Module

Introduces BiLSTM units (hidden_size = 64) to establish cross-timestep feature correlations via gating mechanisms. A dimension permutation operator (Permute) is specifically designed to adapt the CNN feature map format into a sequence vector format, enabling the bidirectional network to fuse both forward and backward propagation context information. Output dimension: (batch_size, L1, 128), where the 128-dimensional feature vector contains concatenated bidirectional hidden states.

High-level Feature Fusion Module

Utilizes depthwise separable convolution (Conv1D: kernel_size = 4, padding = 1) for spatial reorganization of context-rich features, combined with Adaptive Global Average Pooling (AdaptiveAvgPool1d) to extract translation-invariant features. Finally, overfitting is suppressed via a Dropout layer (*p* = 0.3), and classification decisions are made by a fully connected layer.

#### 2.2.4. Data Post-Processing Mechanism

Considering the inertial characteristics of target motion, which tends to maintain its original trend over short periods, the bearing angle change exhibits a continuous and smooth transition on the BTR, without abrupt trajectory bends. This principle provides important theoretical grounding for post-processing algorithm design.

After the neural network switches to tracking mode, for the initial snapshot, the beam with the highest probability of label 1 across all beams is identified as the current target position. Subsequently, the search range for the current snapshot is constrained based on the beam position of the previous snapshot. In this study’s experiments, the target search range for the next snapshot is strictly limited to ±5 beams around the previous target beam position. This history-based search range constraint method reduces the impact of interfering targets on the current tracking target and avoids excessive jumps or unreasonable beam selections.

## 3. Results

### 3.1. Experimental Environment

Experiments were conducted on a high-performance computing platform with dual NVIDIA RTX A6000 GPUs and an Intel Gold 6326 CPU (Santa Clara, CA, USA), paired with 256GB RAM. The software environment was built on Python 3.8 and PyTorch 1.13.0. Key algorithm parameters were set as shown in Table 1:

### 3.2. Simulated Data Validation

**Figure 4:**
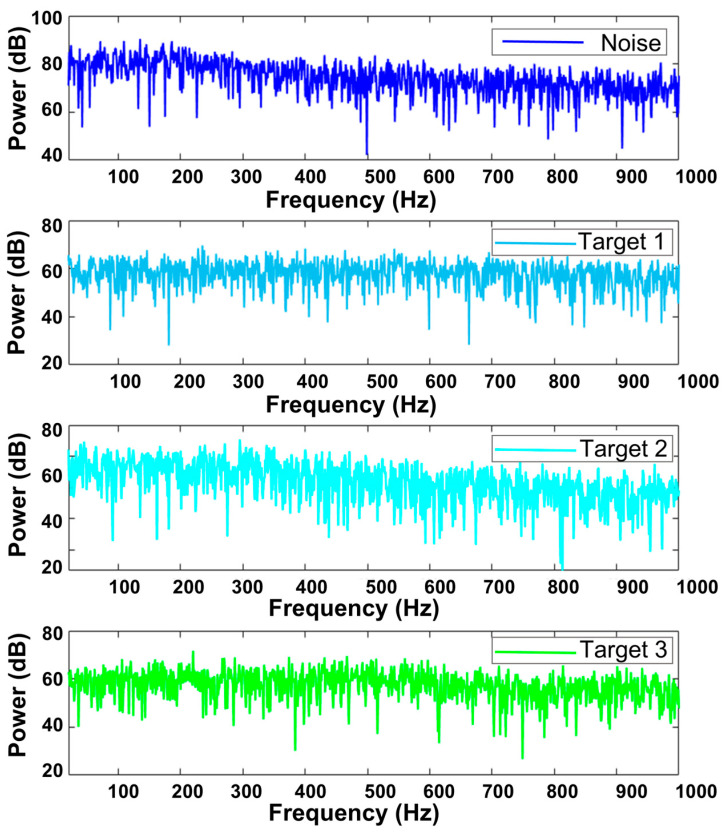
Power spectrum.

**Figure 5:**
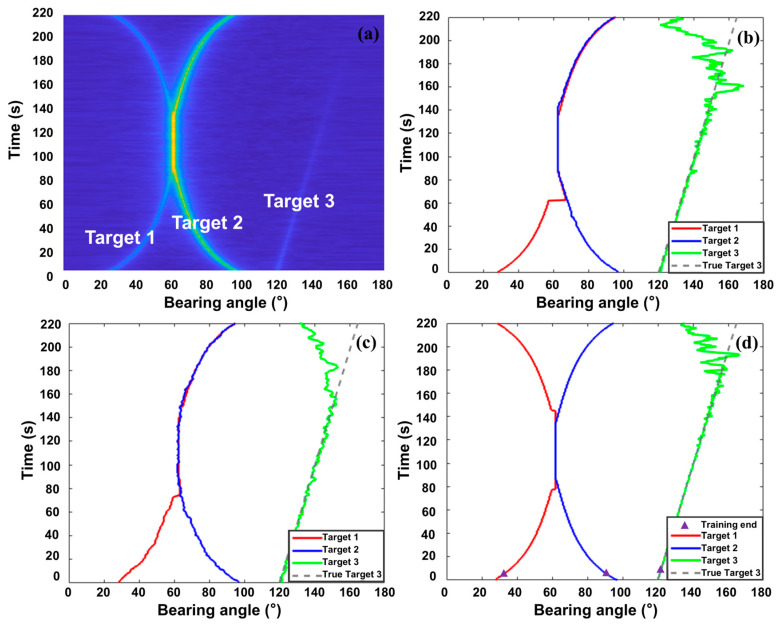
(**a**) BTR; (**b**) Maximum Amplitude Tracker tracking result; (**c**) Particle Filter Tracker tracking result; (**d**) proposed method tracking result.

In the simulation, three moving targets were defined over an observation time T of 220 s. Target 1 starts at 27°, and Target 2 starts at 97°. These targets move towards each other, their trajectories overlapping starting at 80 s and separating after 60 s. This X-shaped trajectory is a primary cause of mis-tracking in multi-target scenarios. Target 3 moved from 120° to 165°, while its energy decayed at a constant rate $k=1-0.9*\frac{t}{T}$, testing the tracking method’s performance under extremely low SNR. The background noise and target signal characteristics are listed in Table 2; their power spectra are shown in Figure 4. The array sampling frequency is 5000 Hz. Each frame uses 1.6 s of data with 80% overlap between consecutive frames. FFT length is $2^{13}$. Conventional beamforming was performed within the 20 Hz to 1000 Hz band; the resulting BTR is shown in Figure 5a.

The proposed method tracked these three targets. Batches of 10 frames were used for training. Target 1 and Target 2 initiated autonomous tracking using two training batches (20 frames); Target 3 required three batches (30 frames). Tracking performance was compared against the Maximum Amplitude Tracker and Particle Filter Tracker. Figure 5b–d show tracking results for the Maximum Amplitude method, Particle Filtering method, and the proposed method. Table 3 quantitatively compares the tracking errors when targets were stably tracked.

Analysis of the simulation results (Figure 5b–d) reveals that during the prolonged trajectory crossing of Targets 1 and 2, both the Maximum Amplitude and Particle Filter trackers incorrectly associated the weaker Target 1 with the trajectory of the stronger Target 2, demonstrating mis-tracking. Furthermore, Table 3 shows the average tracking errors (calculated only before trajectory loss or mis-association) for the three methods. Significant deviation in Target 1′s trajectory tracked by the Particle Filter Tracker is evident even before the crossing, with an average error of 1.38°. In contrast, the proposed algorithm successfully tracked both Target 1 and Target 2 throughout the scenario while maintaining high accuracy, with an average tracking error below 0.2°, highlighting its superiority in handling multi-target tracking.

To examine the minimum SNR required for stable tracking, the stable tracking duration for Target 3 was recorded. The Maximum Amplitude Tracker lost Target 3 after 153 s due to excessively low SNR, followed by the Particle Filter Tracker at 155 s. The proposed method stably tracked until 174 s. Comparing the SNR at the point of loss: Maximum Amplitude failed at SNR < −29.46 dB; Particle Filter Tracker failed at SNR < −29.69 dB; the proposed method failed at SNR < −31.79 dB. This represents a reduction of over 2.1 dB in the minimum required SNR, demonstrating improved detection capability under low SNR conditions. Tracking errors during stable tracking of Target 3 were also recorded. The Particle Filter Tracker exhibited the largest error, although its error for Target 3 (linear trajectory) was slightly lower than for Target 1 (highly maneuvering), which the Particle Filter Tracker struggled to fit. The Maximum Amplitude method stabilized around 0.5° error for Target 3. The error of the proposed method was significantly lower than both, reduced by 32% compared to the Maximum method and 44% compared to the Particle Filter Tracker, demonstrating excellent tracking performance. This stability under low SNR primarily benefits from the model’s global temporal dependency modeling capability.

### 3.3. Sea Trial Data Validation

**Figure 6:**
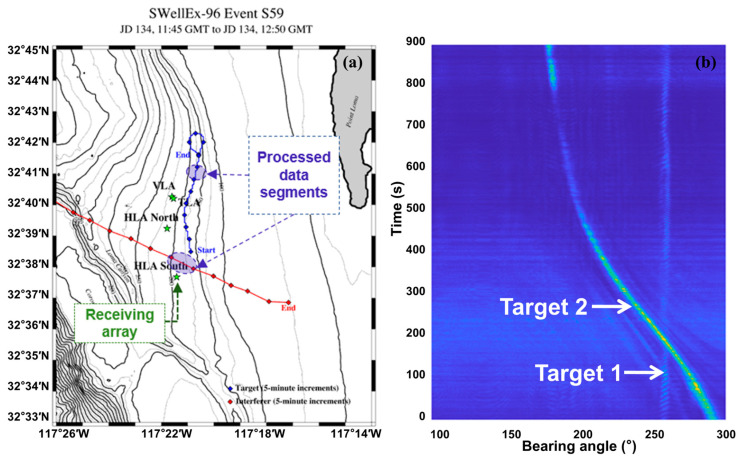
Bearing-bsed target tracking: (**a**) target trajectories; (**b**) BTR.

Experimental data originates from the South Horizontal Line Array (HLAS) data collected during the SwellEx-96 sea trial. The S59 event, containing strong interference sources, was selected for validation. This event is particularly suitable for studying algorithm performance in locating quiet targets amidst strong interference. Target 1 (source ship) started between two horizontal arrays, moving slightly east initially, then proceeded roughly north along the 180 m isobath at 5 knots (≈2.5 m/s). In the last 25 min, the tow ship headed towards port and completed a loop. Target 2 (interferer) started west of all arrays, moved southeast, and ended east of the arrays. Figure 6a shows the target trajectories. A segment containing a trajectory crossing was selected to rigorously validate the proposed method’s performance under multi-target crossing and low SNR conditions. HLAS sampling frequency was 3276 Hz. FFT length was 2048. Frame duration was 0.625 s. Conventional beamforming was applied within the 20 Hz–1000 Hz band over 0°–360°. The BTR in Figure 6b further reveals the complexity: Target 1 itself is weak and severely affected by environmental noise between 100 s and 500 s, coinciding with the crossing of the strong interferer’s trajectory. Signal 2′s energy weakens after 500 s, exhibiting trajectory fragmentation around 700 s.

**Figure 7:**
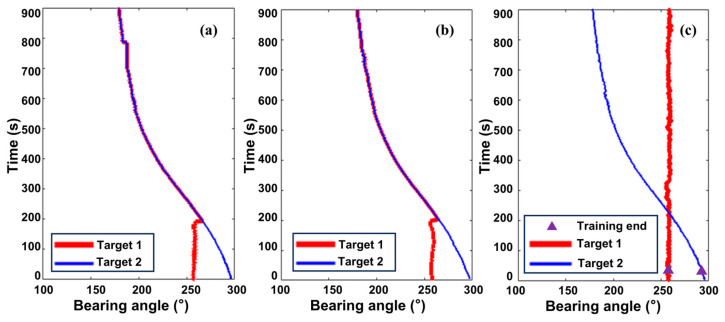
Tracking result comparison: (**a**) Maximum Amplitude Tracker; (**b**) Energy-Based Particle Filter Tracker; (**c**) proposed method.

The proposed method tracked both targets. Targets 1 and 2 initiated autonomous tracking using six training batches (60 frames). Performance was compared against the Maximum Amplitude Tracker and Particle Filter Tracker, with results shown in Figure 7.

Analysis of Figure 7a (Maximum Amplitude) shows that at the trajectory crossing, the weak target (Target 1) is mis-associated with the trajectory of the strong target (Target 2), illustrating the method’s limitation with crossing targets. Furthermore, when Target 2′s energy weakens, causing temporary disappearance, the Maximum method fails to locate it until energy increases again. Figure 7b (Particle Filter Tracker) shows that, unlike the Maximum method, it maintains track continuity during temporary trajectory fragmentation, demonstrating an advantage. However, at the crossing point, the Particle Filter Tracker also suffers mis-association, linking the weak target to the strong target’s trajectory. In contrast, the proposed method, shown in Figure 7c, effectively overcomes the deficiencies of the other two methods. It successfully distinguishes between the targets after crossing and separation, maintaining good accuracy throughout the tracking process. These results fully demonstrate the superiority of the proposed method in handling multi-target tracking challenges.

### 3.4. Computational Complexity Analysis

Neural network methods typically entail higher computational overhead compared to traditional approaches. To alleviate this computational burden, this study enhances practical feasibility and real-time performance through model lightweighting and inference process optimization. The complexity of the proposed tracking method primarily stems from two components: neural network forward inference and data post-processing. The forward inference stage employs a streamlined network architecture, while the post-processing stage incorporates the local optimization principle of the maximum value method. To quantitatively evaluate the computational efficiency of the proposed method in practical operation, we measured the average processing time per frame for three methods under the same experimental conditions as those described in Section 3.3 for simulated experiments, using a single target sequence spanning 680 frames. The results are shown in Table 4 and Table 5.

Experimental results show that the proposed neural network-based tracking method is admittedly slower in computational speed compared to conventional methods. Notably, however, its per-frame processing latency is maintained within the millisecond regime, making it compatible with the frame rate requirements of most real-time systems. Furthermore, it can be observed that the computational overhead of the neural network method primarily stems from the forward inference process.

## 4. Discussion

This study has not only proposed an innovative deep learning-driven underwater multi-target tracking algorithm at the technical level, featuring on-site training and the fusion of spatiotemporal feature learning with physical motion constraints, but also demonstrated its significant practical effectiveness through simulation and SwellEx-96 sea trial validation: (1) improved tracking accuracy under trajectory crossing scenarios; (2) enhanced tracking performance under low SNR conditions. This work enriches research on deep learning techniques in underwater target tracking and provides robust technical support for the autonomous navigation of future underwater unmanned systems.

Although the proposed lightweight neural network-based tracking method currently exhibits lower computational efficiency compared to conventional approaches, it substantially improves tracking accuracy and robustness in challenging environments characterized by low SNR and multi-target crossing scenarios. Through structural lightweight design and inference optimization, the method achieves an effective balance between accuracy and complexity.

With ongoing advancements in GPUs, TPUs, and specialized neural processing units—particularly the rapid improvement in computational power of edge devices—the time cost of neural network inference is decreasing rapidly. Therefore, the current limitations in computational efficiency are expected to be mitigated in the near future, making the proposed approach more applicable and feasible for next-generation underwater intelligent tracking systems.

In summary, the proposed underwater multi-target tracking algorithm is a promising approach, but opportunities for improvement remain in algorithm optimization to enhance its efficiency and applicability.

## Figures and Tables

**Table 1:** Key algorithm parameters.

Parameter	Value
Initial Learning Rate	0.001 (Adam Optimizer)
Training Epochs	50 epochs
Number of Particles	500
Search Window	±5 beams
Decision Threshold	0.5 beams
Simplified Features	32 features

**Table 2:** Signal Characteristics.

Signal Name	Signal Type	Spectral Characteristics	SNR
Background Noise	Continuous Spectrum (Isotropic)	No attenuation 20–200 Hz, −5 dB/oct above 200 Hz	/
Target 1 Signal	Continuous Spectrum + Line Spectrum (400 Hz)	No attenuation 20–600 Hz, −6 dB/oct above 600 Hz	−18.0 dB
Target 2 Signal	Continuous Spectrum + Line Spectrum (200 Hz)	No attenuation 20–300 Hz, −6 dB/oct above 300 Hz	−13.0 dB
Target 3 Signal	Continuous Spectrum + Line Spectrum (120 Hz, 220 Hz)	No attenuation 20–500 Hz, −6 dB/oct above 500 Hz	Reduce from −21.0 dB to −40.3 dB

**Table 3:** Comparison of average tracking errors across different methods.

	Target 1 Error	Target 2 Error	Target 3 Error
Maximum Amplitude Tracker	0.54°	0.52°	0.50°
Particle Filter Tracker	1.38°	0.63°	0.61°
Proposed Method	0.18°	0.17°	0.34°

**Table 4:** Average single snapshot processing time for different tracking methods.

Maximum Value Tracking Method Average Frame Time (seconds)	Particle Filtering Method Average Frame Time (seconds)	Neural Network Method Average Frame Time (seconds)
2.00 × 10^−6^	1.68 × 10^−4^	2.09 × 10^−3^

**Table 5:** The time decomposition and training cost of neural network method.

Training Total Duration (seconds)	Tracking Total Time (seconds)	Forward Propagation Time (seconds)	Post Processing Time (seconds)
2.39	1.4467	1.4249	0.0205

## Data Availability

The sea trial data used in this study come from the SwellEx-96 experiment. This dataset is already available at https://swellex96.ucsd.edu/index.htm, publicly released. Due to the incomplete status of related research within the group, it is temporarily not convenient to disclose the relevant code. If there is a need, it can be obtained from the corresponding author through reasonable requests.
